# Evaluating the Distribution, Quality, and Educational Value of Videos Related to Knee Instability Exercises on the Social Media Platform TikTok

**DOI:** 10.7759/cureus.57104

**Published:** 2024-03-28

**Authors:** Brandon D Rust, Elie Christoforides, Ambika Singh, Simon Wahba, Jashkumar Choudhari, Jackson Copper, Aidan Kaspari, Vijay Patel, Santiago Ortiz, Desiree E Ojo, Khavir A Sharieff

**Affiliations:** 1 Osteopathic Medicine, Nova Southeastern University Dr. Kiran C. Patel College of Osteopathic Medicine, Fort Lauderdale, USA; 2 Osteopathic Medicine, University of the Incarnate Word School of Osteopathic Medicine, San Antonio, USA; 3 Surgery, Nova Southeastern University Dr. Kiran C. Patel College of Osteopathic Medicine, Tampa, USA

**Keywords:** orthopedic sports medicine, knee instability, patient education, tiktok, social media

## Abstract

Introduction

TikTok, a globally popular short-form video platform, offers a unique space for healthcare professionals to share advice, particularly under common conditions such as knee pain or instability. Despite its popularity, doubts persist regarding the reliability of medical information disseminated on TikTok. This study aimed to evaluate the quality of TikTok videos as a source of patient information on knee instability, recognizing the need for a comprehensive assessment of potential misinformation on this influential social media platform.

Methods

A search for “knee stability exercises” on TikTok yielded 448 videos, of which 187 met the inclusion criteria. These videos were categorized by source and evaluated using the Knee Exercise Education Scoring Tool (KEEST) and an information analysis questionnaire, DISCERN.

Results

General user videos (69.84%) had notably lower DISCERN scores than healthcare professional videos (29.1%) across all categories (P < 0.001, P = 0.282, P = 0.131, and P = 0.010). The DISCERN scores were inversely linked to video metrics (views, likes, comments, favorites, and shares). General user videos were largely of poor quality (66.4%), whereas healthcare professional videos spanned poor (61.8%), fair (28.2%), good (9.1%), and excellent (1.8%) categories. Both general users (12.31/25) and healthcare professionals (12.18/25) exhibited average quality according to KEEST standards (P = 0.809), with an intriguing inverse correlation between video popularity and DISCERN score.

Conclusion

Healthcare professionals demonstrated superior evidence-based content (DISCERN), whereas both groups were comparatively educated on treatment plans and effects (KEEST). TikTok’s prevalent knee instability videos lack quality, proper sourcing, treatment risk information, and explanation. Moreover, popularity is inversely correlated with quality, and healthcare professionals appear to offer better evidence-based content. TikTok’s role in healthcare highlights the importance of ensuring accurate information and implementing content quality regulations.

## Introduction

In the United States, approximately 80% of the population engages in Internet-based social networking, representing a substantial target demographic for the dissemination of medical guidance [1]. TikTok, a short-form video-based platform, is one of the most widely used social networking services worldwide because of its broad range of content, convenient access, and user-targeted algorithmic ability to bridge gaps between various communities [2,3].

Moreover, healthcare professionals utilize TikTok to offer medical guidance, leveraging the platform’s accessibility to share general advice on common conditions, such as knee pain. Knee pain may result from misalignment or a lack of stability during joint articulation [4]. The prevalence of knee pain is noticeably increasing, with approximately 65% of individuals experiencing it and approximately 25% of adults in the United States experiencing various forms of knee-related discomfort. In 2018, knee pain prompted approximately four million annual primary care visits [5]. Although over 50% of physicians and nurses endorse online patient education for general conditions, a limited percentage of these professionals express confidence in the reliability of such information [6].

Several studies have explored the reliability of medical advice disseminated through social media platforms, encompassing diverse health topics. For instance, one study found that the educational value of medical advice conveyed through TikTok videos regarding anterior cruciate ligament (ACL) rehabilitation exercises was generally poor. In the hope of finding an explanation for their conclusion, the authors found that the short video playtime and engagement times on TikTok might not allow for an adequate understanding of the medical advice being conveyed [7]. In another study assessing the quality and educational benefits of TikTok videos related to Achilles tendinopathy, researchers found that videos focused on Achilles tendon exercises were of lower quality and reliability [8]. Another study on TikTok revealed that 90% of the content addressing prevalent orthopedic conditions emanated from non-physician sources, which underscores the scarcity of guidance directly from medical professionals [1].

The intersection of TikTok’s role as a platform for healthcare advice and the escalating prevalence of knee pain underscores the imperative need for comprehensive studies exploring the potential for misinformation on proactive knee care disseminated through this social media platform. This study aimed to evaluate the quality of TikTok videos as a source of patient information on knee instability.

## Materials and methods

Search strategies

**Figure 1:**
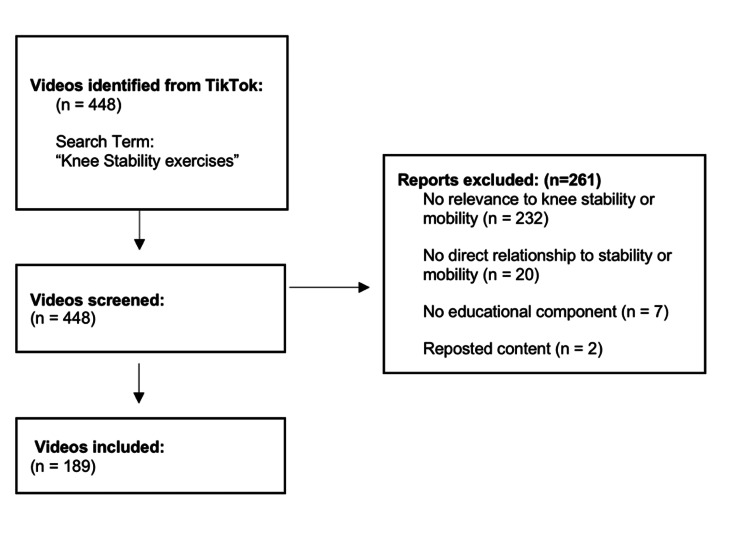
Flowchart of the search process for videos related to knee instability exercises

The social media app TikTok was searched on June 20, 2023, for videos pertaining to knee stability exercises. The search results for the key term “knee stability exercises” produced 448 results without search filters. This helps populate videos during the initial search that general TikTok users would most likely encounter first when searching for knee stability exercises. It is important to note that TikTok states that search results are partially influenced by users’ previous engagement, including views, likes, comments, and shares. Therefore, search results may vary depending on general users’ (layperson) previous engagement with the app [9]. After the initial search, screening was performed initially by two authors (VP and SO). When different groupings were obtained for the same video, discrepancies were resolved by a third author with relevant experience in exercise education as a certified personal trainer (BDR). Videos were excluded due to (1) having no relevance to knee stability, pain, mobility, or strengthening (n = 232); (2) exercises not directly related to knee stability or mobility (n = 20); (3) videos with no educational component (n = 7); and (4) reposted content (n = 2). After screening, 189 videos remained for data analysis (Figure 1).

For each video included in the analysis, the username and number of views, likes, comments, and favorites were documented. A link to the TikTok video was recorded, along with the date on which it was posted. The authors also recorded whether there was a scientific literature citation in the description or the video itself. Finally, videos were separated into two categories based on whether a healthcare professional or general user posted them. User profiles, video introductions, and degrees of evidence were examined to create this designation. The healthcare professional category included videos of physicians, physical therapists, nurses, chiropractors, or any other licensed professional with relevant knowledge in the field. Because there were no human participants in this study, ethics committee approval was not required.

DISCERN for reliability and quality assessment

The DISCERN questionnaire, developed in the late 1990s, is a valuable tool for consumers to assess the quality of health information in published studies. With 16 questions organized into three categories, DISCERN evaluates the reliability of publications (DISCERN 1), the quality of information on treatment choices (DISCERN 2), and overall publication quality as a source of treatment information (DISCERN 3). Each question is scored from one to five, with a cumulative score that determines the quality rating, ranging from excellent (63-75 points) to very poor (16-26 points). This well-validated tool offers a comprehensive assessment of treatment information, considering factors such as reliability and overall quality, and can be used to judge the quality of a publication without the need for specialist knowledge [10,11].

Knee Exercise Education Scoring Tool (KEEST)

KEEST, designed to assess the educational suitability of videos, is a modified tool derived from studies of scoliosis exercises and shoulder instability videos [7,12]. Comparing five questions related to the exercise cycle, target area, expected effect, precautions, and rationale, the scoring system ranges from 0 to 5 for each question, indicating increasing quality with higher scores. The final KEEST score, ranging from 0 to 25, reflects the overall quality of the video’s educational content. This tool provides a concise and standardized evaluation, enabling a systematic assessment of the educational value of videos.

Assessment and scoring criteria

For data analysis, two separate scoring systems were utilized to evaluate the quality and educational value of the chosen videos: the DISCERN questionnaire was used to assess the reliability and quality of the treatment described in each video, and the KEEST was used to evaluate the educational suitability of each video.

The videos were independently evaluated by three authors trained in both scoring systems (EC, JC, and BDR). Training consisted of example videos that ranged from low to high scoring on both scoring systems to understand what scores and values should be attributed to what content and to attempt to standardize scores overall. Disagreement between evaluators was resolved by discussion, and a highly effective interrater agreement was reached (r > 0.82).

Analysis

A statistical analysis was conducted to elucidate the scoring and characteristic data of the examined variables. Descriptive statistics, including mean, SD, median (IQR), and percentages, were used to summarize the key features of the data. For the fundamental characteristics of the analyzed videos, median (IQR) values were reported instead of means to mitigate the influence of outliers within the data on the outcomes. To ensure robustness, Levene’s test was additionally employed to confirm the suitability of the two-sample t-test, which was used to compare the two types of uploaders based on the mean, SD, and sample size of each continuous and categorical variable. The threshold for statistical significance was set at P < 0.05 for all comparisons. All statistical analyses were performed using IBM SPSS Statistics for Windows, Version 27.0 (Released 2020; IBM Corp., Armonk, NY, USA) and Python, Version 3.11 (Released 2022; Python Software Foundation, Wilmington, DE, USA) to enhance the reliability and comprehensiveness of the results.

## Results

Video characteristics

Out of 448 videos generated by the search term “knee stability exercises,” 187 met the inclusion criteria and were included in the final analysis. The median views were 9,195 (IQR = 943.25-67,950), likes 356 (IQR = 48-3,273), and the median for comments, favorites, and shares were 3 (IQR = 1-29), 104 (IQR = 14-1,134), and 27 (IQR = 2-248), respectively (Table 1).

**Table 1:** Characteristics of included videos

Characteristics, median (IQR)	Included videos (n = 187)
Number of views	9,195 (943.25-67,950)
Likes	356 (48-3,273)
Comments	3 (1-29)
Favorites	104 (14-1,134)
Shares	27 (2-248)
Scoring, mean (SD)	
DISCERN 1	20.44 (3.62)
DISCERN 2	13.91 (4.51)
DISCERN 3	2.20 (0.94)
Total DISCERN	36.56 (7.82)
KEEST	12.28 (4.92)

Types of uploaders

The upload frequency was higher among general users (n = 132, 70.6%) than among healthcare professionals (n = 55, 29.4%). Statistical analysis revealed no significant differences in the observed characteristics between general users and healthcare professionals (Table 2).

The overall DISCERN score exhibited statistical significance (P = 0.010), with the DISCERN 1 score, in particular, demonstrating notable statistical significance (P < 0.001) in the comparison between healthcare professionals and general users. In contrast, DISCERN 2 and DISCERN 3 did not attain statistical significance (P = 0.282, P = 0.131). KEEST scores, on a 25-point scale, were comparable between the two groups: 12.31 for general users and 12.18 for healthcare professionals (P = 0.809) (Table 2).

**Table 2:** Characteristics of videos across the two types of uploaders

Characteristics, median (IQR)	General users (n = 132)	Healthcare professionals (n = 55)	P
Number of views	13,500 (1,245.75-94,350)	3,003.5 (668.25-22,075)	0.149
Likes	529 (64-4,511.5)	94 (40.5-1,655)	0.211
Comments	9.5 (1.5-39)	4 (0-14)	0.201
Favorites	224 (20-2,072)	31 (9.5-310)	0.167
Shares	37 (3-304)	5 (1-65.5)	0.013
Scoring, mean (SD)			
DISCERN 1	19.99 (3.57)	21.55 (3.52)	<0.001
DISCERN 2	13.75 (4.40)	14.3 (4.76)	0.282
DISCERN 3	2.16 (0.93)	2.32 (0.96)	0.131
Total DISCERN	35.90 (7.63)	38.16 (8.06)	0.01
KEEST	12.31 (4.95)	12.18 (4.85)	0.809

Table 3 illustrates the DISCERN grading outcomes, revealing comparable scores across all the categories. General users had a higher proportion of videos graded as very poor (27.2%) than did healthcare professionals (20.0%). Conversely, healthcare professionals produced a greater number of videos graded as poor (40.9%) than did general users (39.6%). In the fair category, healthcare professionals had a higher percentage (28.2%) than did general users (23.5%). Notably, general users had a higher proportion of videos graded as good (9.7%) than did healthcare professionals (9.1%). However, healthcare professionals had only a small number of videos graded as excellent (1.8%), whereas general users had none (0%).

**Table 3:** Percentage of DISCERN grades across the two types of uploaders

Grading	General users	Healthcare professionals	Total
Very poor	27.2%	20.0%	25.1%
Poor	39.6%	40.9%	39.9%
Fair	23.5%	28.2%	24.9%
Good	9.7%	9.1%	9.5%
Excellent	0.0%	1.8%	0.5%

## Discussion

This study identified discrepancies in the available posts between medical professionals and general users on TikTok related to knee stability exercises. With over 116,000 views on 187 videos, our findings revealed that more than 75% of the views were attracted to content created by general users, highlighting significant variability in material quality and an increased risk of misinformation dissemination. The analysis indicated that videos by healthcare professionals had higher DISCERN scores, demonstrating better quality than those by general users; however, paradoxically, videos with lower DISCERN scores garnered more engagement on the platform. The overall quality of the videos provided by the KEEST scores was not significantly different between the two groups.

Our findings showed that general users exhibited significantly lower DISCERN scores in all categories. Conversely, healthcare professionals exhibited a notable improvement in grading scores, with a majority being assessed as “fair” and “good,” and some even receiving the distinction of “excellent” (35.9 versus 38.6, P = 0.010). Healthcare professionals created content that was of higher quality than that of general users. Moreover, the KEEST scores displayed statistical insignificance, meaning that healthcare professionals exhibited a similar degree of describing the benefits and risks of an exercise while acknowledging shortcomings for knee treatment options (12.31 versus 12.18, P = 0.809). Other studies have used the DISCERN scoring tool to grade the quality of educational TikTok videos. The current literature covers a variety of orthopedic-related topics, including those related to scoliosis and ankle and shoulder stability strengthening exercises [8,12,13]. Similar to our results, all studies revealed a disparity in the quality of rehabilitation exercises between general users and healthcare professionals, emphasizing the need for improved scientific regulation [8,12,13].

In addition, videos with lower DISCERN scores garnered more favorites, likes, views, comments, and overall engagement due to potential issues with the reliability and quality of health information about treatment choices. Repeated engagement in exercises with poor overall quality may worsen instability, increase muscular imbalance, or exacerbate prior injuries, especially if eccentric loading is improperly performed [14]. This paradoxical trend underscores the need for the increased involvement of healthcare professionals in addressing the dissemination of inaccurate medical information on these platforms. Yoo et al. reported similar findings on the suitability of videos for enhancing knee stability on YouTube, discouraging its use as a source for learning and emphasizing that professionals, particularly those in rehabilitation medicine, should prioritize uploading high-quality videos with reliable content on the platform [15]. Kolade et al. investigated the popularity and accuracy of orthopedic content on TikTok and Instagram, which revealed a significant prevalence of misinformation across common conditions such as tennis elbow, rotator cuff, Achilles tendon, ACL, meniscus, and ankle ligament tears [1]. In contrast, Kim et al. argue that the public can access valuable, reliable, and informative therapeutic information on social media platforms [16]. These findings may be influenced by differences in diseases or the complexity of the covered content, emphasizing the nuanced nature of evaluating the educational material on the platform.

Creators can enhance the quality of their videos by attaching literature, references, and additional links to the video descriptions, providing viewers with access to more in-depth information. This not only enriches educational value but also contributes to a more informed audience. Although infrequently utilized, educational posts are positively received by the orthopedic community, proving that TikTok can also implement content validation tools that would introduce a peer-review element to enhance accuracy and reliability [17]. Recognizing TikTok’s potential as a potent tool for content sharing, especially within the healthcare domain, underscores the need for careful consideration of information accuracy and the introduction of regulatory measures to uphold content quality standards.

Several limitations of our study should be acknowledged when interpreting the findings. This study introduced a selection bias in the search terms used. We opted to utilize a comprehensive search term, “knee stability exercises,” to replicate a query that a layman user interested in the subject matter might employ to access relevant videos. Nevertheless, individuals familiar with a specific subcategory of knee instability may choose to employ a more personalized and nuanced search term. Moreover, TikTok’s algorithm, which is employed in a video population under a consistent search term, may yield varied results contingent on the user’s prior interactions within the application. Additionally, the grading tools employed in this study inherently encompass an element of subjectivity in evaluating the content quality. To mitigate this potential bias, both the DISCERN and KEEST tools were used. However, factors such as personal judgment and subjective perspectives may contribute to variations in scoring, highlighting the inherent challenge of achieving complete interobserver agreement.

## Conclusions

This study scrutinized the quality of knee instability related to TikTok videos, uncovering a significant scarcity in quality, a concerning pattern in which inferior videos gain more attention, and deficiencies in sourcing, treatment risk information, and explanations. Healthcare providers should recognize the widespread dissemination of such content and promote awareness of the platform’s educational limitations in delivering medical information.
